# Transmission Power Influence on WSN-Based Indoor Localization Efficiency

**DOI:** 10.3390/s22114154

**Published:** 2022-05-30

**Authors:** Amr Elkenawy, Janis Judvaitis

**Affiliations:** Institute of Electronics and Computer Science, 14 Dzerbenes St., LV-1006 Riga, Latvia; janis.judvaitis@edi.lv

**Keywords:** indoor localization, WSN, RF, range-based, RSSI, multilateration, WLS, PDR

## Abstract

Performing a good-quality indoor localization of a mobile target is a challenging task, which can be affected by many factors such as radio wave behavior, the nature of the experimental environment, and available infrastructure. (1) Background: An indoor localization experiment using an Internet of Things (IoT) wireless sensor network (WSN) testbed is performed, in order to study the influence of transmission power level on the quality of received signals, and consequently, the estimated target positional coordinates. (2) Methods: A received signal strength indicator (RSSI) range-based localization system using a geometrical constrained weighted least squares (WLS) estimator multilateration technique is selected to validate the influence of the transmission power level on the performance of the localization algorithm. (3) Results: Fair localization quality was obtained at the highest transmission power level instead of the proposed transmission power level. (4) Conclusion: Additional factors are discussed to fully represent the required operational transmission power for a better localization quality, along with suggested improvements of the infrastructure configuration as a future work.

## 1. Introduction

Localization of objects inside an indoor environment is emerging as an important requirement in many fields and industries. The definition of localization is to estimate the positional coordinates with a sufficient certainty, with respect to the dimensions and nature of a defined environment. Example applications of indoor localization could be localizing a person in a room, a forklift within a warehouse, or a mobile agent within a laboratory. Localization is the first and principal step of the see–think–act cycle for tasks to be executed by any mobile agent [1]; for example a forklift driver navigating the warehouse to reach a destination point while being aware of its current location or a medical staff localizing a patient in a hospital as a part of a patient surveillance system. Another large and fast growing domain that will take advantage of indoor localization is human–robot collaboration [2], whereby the accurate localization of a moving human worker might provide additional safety and efficiency benefits if integrated correctly.

Although the global navigation satellite system (GNSS) is a well-known absolute location system for localization in a wide variety of applications, the GNSS does not provide a reliable signal for indoor environments, because of the abundance of barriers and obstructions inside such environments [3]. A popular example of GNSS would be the global positioning system (GPS), which uses a shared frame of reference for all located objects (GPS receiver uses longitude, latitude, and altitude); on the other hand, a relative location system would use its own frame of reference [4]. The radio frequency (RF)-based localization algorithms can be classified into two basic models: range-based and range-free models. Both models rely on an infrastructure of anchor nodes, transmitting/receiving beacons, installed at fixed spatial locations within the environment for the sake of localizing a mobile target node. Range-free models assume no prior knowledge about RSSI signal strength, which means no initial calibration of anchor nodes is required, in contrast to range-based models, which need both signal strength and location calibration of anchor nodes [5].

Indoor localization systems can be categorized according to their measurement parameter such as received signal strength indicator (RSSI), time of arrival (ToA) and angle of arrival (AoA). The RSSI technique is a popular option because it is easy to implement and needs no specific hardware [6].

The relative distance information between transmitter and receiver can be extracted from the RSSI value by using a mathematical formula mapping the RSSI value to a relative distance, known as radio channel model or path-loss model [7]. In this particular indoor localization problem, the location of a target or an object is represented as an absolute position within the room frame of reference, which is obtained by using several relative distances between the mobile target and a fixed number of anchor nodes. The algorithm responsible for translating a set of relative distances between anchor nodes and mobile nodes to an absolute position of the mobile node could be multilateration (or trilateration when the number of anchor nodes is 3) [8].

The radio transceiver is an embedded electronic chip inside the transmitter/receiver device responsible for sending and receiving radio packets from/to the target to be localized (the direction of packets depends on the system’s topology). In case of a successful reception of a radio packet, the power of the packet can be represented as a RSSI digital value, stored in a built-in register of the radio transceiver [9].

### 1.1. Related Work

Although the RSSI is widely used as a core variable in indoor localization applications due its simplicity and hardware availability, it is constantly prone to multiple sources of error and could be easily affected by environmental noises.

RSSI-based localization algorithms have evolved over time in order to mitigate the effects of various sources of noise on the localization quality. The classical localization range-based systems, relying mainly on the path-loss radio channel model and multilateration localization algorithm could be used as a baseline to survey the evolution of the available RSSI localization approaches. The path-loss model is used to estimate the relative distances between transmitters and receivers [10], which can be further translated into an absolute distance of the target node by using any localization algorithm such as the multilateration algorithm or the min-max algorithm [11]. Range-based models usually provide a poor description of the environment in terms of flexibility and precision due to the negative effects of multi-path and non-line-of-sight propagation, which in turn degrades the localization accuracy [6].

Another category of advanced localization models includes range-free models and time-based techniques, which are considerably researched compared to the range-based models [12]. The range-free models or fingerprinting relies on a fingerprint database or a radio map, consisting of radio wave signatures gathered from several anchor nodes or access points at reference positions throughout the area of interest [13]. Although fingerprinting models try to ensure a higher accuracy in various environmental conditions, it requires a special environmental survey for each deployment environment, and it still suffers from RSSI fluctuations, signal interference, and multi-path fading. The time-based techniques can be used to overcome some of the fingerprinting model drawbacks by utilizing the signal’s propagation time and velocity, but additional stages of filtering or map matching are required in order to improve the localization performance [12].

Beside the well-known deterministic approaches discussed above, probabilistic or machine-learning solutions proposed in [14] could be applied to this localization problem. Ref. [15] proposes a statistical (recursive) algorithm that outperforms the conventional algorithms of Kalman and particle filters for both static and dynamic environment use cases. Although the main purpose behind the aforementioned nondeterministic solutions is to mitigate the effect of some noise sources, it will make the existent localization problem more complex. The classical localization range-based systems can be adjusted or augmented in order to increase the localization accuracy by performing various sets of calibration routines or an extensive analysis of radio signal propagation [6]. Another option is to increase the number of anchor nodes so that a greater amount of information is gathered. The interference level between signals would potentially increase as a result, which imposes signal filtering as a must to improve system’s accuracy [16]. The reasonable approach of exclusively tuning the transmission power while preserving the same structure, hardware setup, and calibration routine is proposed by [17] and would be validated and extended in our work due to its uniqueness and simplicity.

The rest of this subsection presents a summary of procedures and work done in [17], which is used as a starting point for our work based on specific experiment conditions, infrastructure, and environment. The purpose of the work done in [17] is to find the optimal transmission power level of the mobile node which increases the accuracy of the overall localization system, by improving the quality of individual relative distances measured by anchor nodes. These relative distances are used as an input to the multilateration algorithm. The RSSI parameter is used as a core variable for distances estimation; that is why a path-loss model from [18] is chosen, to extract distance information out of the RSSI value. The main approach for improving the accuracy is to maximize the system’s overall sensibility according to [17], which in turn allows the detection of smaller variation of distances. A heuristic approach is followed to describe the sensibility of the entire system as a single parameter *N*. A classical multilateration algorithm is used to perform the mobile node localization as a part of a range-based localization system by utilizing a setup of 5 Z1 Zolertia anchor nodes attached to the walls and a single mobile Z1 Zolertia node fixed on a tripod. Six test points in total are recorded, five of which are contributing to the calibration process and path-loss model derivation, while the sixth point is left out for result validation. The calibration step defines the path-loss model of each anchor’s antenna and plays a vital role in figuring out the optimal transmission power of the system by estimating the intrinsic and extrinsic parameters of each anchor’s antenna. The multilateration algorithm is used to estimate absolute position of the mobile target node in six predetermined test positions; then the output results of the algorithm are visualized and evaluated by using mean error and root mean square error (RMSE) metrics. The RMSE value of the validation point (sixth point) is found out to be the lowest, at a certain power level. This power level is directly linked to maximizing the system’s sensibility.

### 1.2. Purpose of Paper

This work is an extension of the work done in [17] with an objective of testing the effectiveness of the power tuning approach for reducing the localization error in RSSI-based localization systems and validating results obtained in the previous research through a detailed experiment executed on the EDI TestBed [19,20]. The EDI TestBed is a 100+ node heterogeneous Internet of Things and wireless sensor network testbed distributed around a 7-floor building, inside and outside for validation and research of Internet of Things and wireless sensor network protocols. Our work aims to present an initial evaluation of the EDI TestBed Infrastructure as a Service (IaaS) provided by the DevOps-ready IoT testbed [21]. We would like to contribute to the current indoor localization research by deploying a geometrical constrained range-based RSSI indoor localization system and investigating power consumption levels for energy-harvesting realization

Relative distances between anchor nodes and the mobile target to be localized are estimated by using a path-loss radio channel model and then fed to the multilateration algorithm responsible for estimating the absolute location of the mobile target. The deployed localization system is intended to verify the work and results of [17], achieving the lowest estimation error by finding the optimal transmission RF output power through tuning the power level of anchor nodes (transmitters). The results of this paper did not completely match the utilized concept of [17] because of different infrastructure setup and environmental factors. Eventually this paper added new criteria to the proposed approach of [17] for obtaining the correct power level and proposed future ideas and improvements. The main design choices of our deployment in addition to the underlying motivation are listed below:

- EDI TestBed workstation anchor nodes are not equipped with external antennas in order to evaluate our existing testbed’s infrastructure readiness for similar experiments;
- the target node acts as a gateway and network sniffer to keep the architecture simple and take into consideration the privacy of location data;
- same-system sensibility criterion as in [17] is selected in order to obtain comparable results for further validation;
- a geometrically constrained multilateration algorithm is utilized to enhance the quality of measurements by means of constraining distance estimations using a descriptive algorithm.

## 2. Materials and Methods

In order to build a complete RSSI range-based indoor localization system, three main components must exist:

1. RF transceivers (anchor nodes and target node);
2. radio channel model;
3. Multilateration localization algorithm.

RF transceivers of anchor or stationary nodes are responsible for sending network packets to the target node, which in turn tries to receive packets correctly and extract the RSSI information out of it. Configuration and internal components of anchor nodes are described in Section 2.1, and hardware/software configuration of the target node (gateway) is described in Section 2.2. The RSSI data is recorded at a number of test points, described in Section 2.3 in order to build radio models of anchor nodes (Section 2.4), describe system sensibility (Section 2.5), and evaluate the overall system’s performance as a result. The final part of the localization system is the multilateration algorithm, which is described in Section 2.6, and then modified/enhanced to include the geometry constraints of Section 2.7.

### 2.1. Anchor Nodes

**Figure 1:**
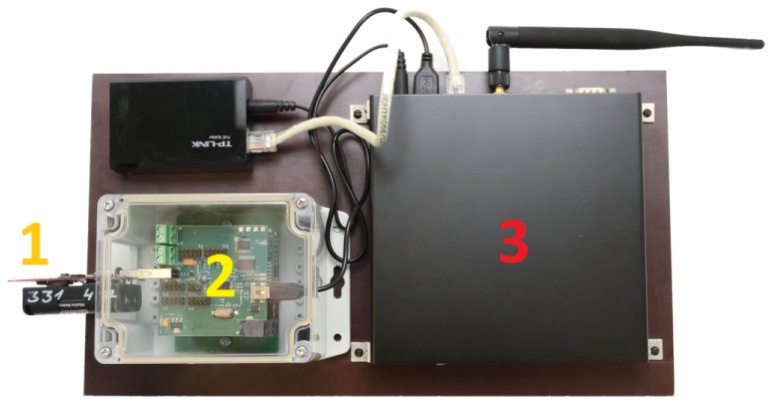
EDI TestBed workstation [19].

Being used as anchor nodes, 4 EDI TestBed workstations connected to XM1000 sensor nodes (called Devices Under Test or DUTs in EDI TestBed) [19] are attached to the ceiling of a 5.8 m × 5.6 m × 2.7 m office room. A complete testbed workstation consists of: (1) DUT, (2) testbed adaptor, and (3) Ethernet router as shown in Figure 1 below.

**Figure 2:**
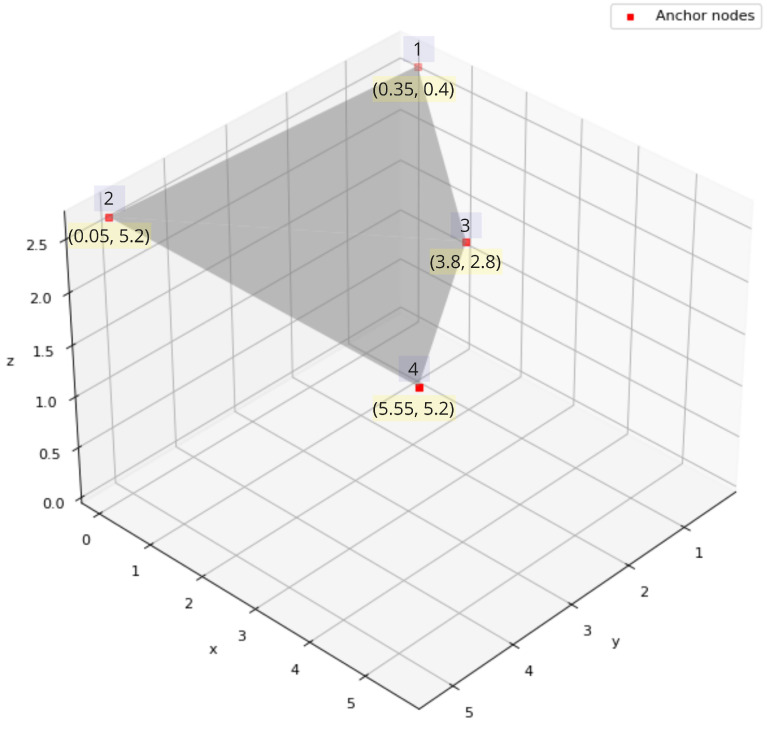
Locations of anchor nodes inside the room.

Locations of workstations are chosen to be at the corners, which is the case for 3 nodes, whereas the fourth node is near the midpoint of the ceiling, being away from any wall is aimed to give more uniform wave propagation toward the target node, while providing an easy access and space for calibration as described in Section 2.4.1. Figure 2 illustrates positional coordinates of anchor nodes with respect to the room environment at a height of 2.7 m (z = 2.7 at the ceiling level).

The anchor nodes send network packets periodically over the air with their information through the CC2420 radio transceiver [9], programmed by using a C program of the MansOS embedded operating system [22] which is flashed on XM1000 sensor nodes. The main content of messages/packets is anchor number (or NodeID, as described in Section 2.2). When the message is successfully received by the target node, the content is parsed. Then the RSSI value is utilized to estimate the relative distance between the sending anchor node and the mobile target to be localized.

### 2.2. Gateway

**Figure 3:**
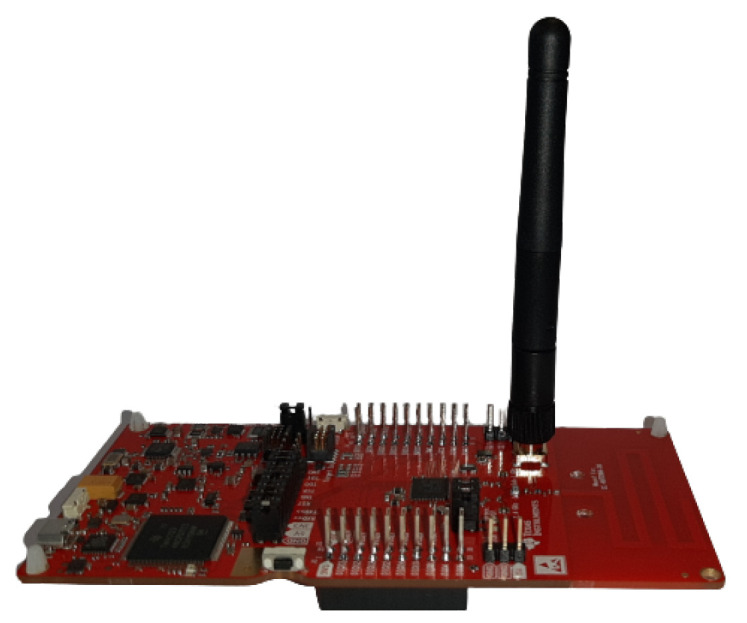
Target node hardware.

The gateway consists of the mobile target node (packet sniffer) connected to a laptop [18]. The main role of the packet sniffer is listening to packets being sent from anchor nodes, transferring them to the computer where the received packets are parsed and processed. The Texas Instrument (TI) LaunchPad CC1352R1 [23] is used as the mobile target node hardware. The Launchpad is equipped with an external antenna (2.4 GHz dipole antenna with 2.8 dBi peak gain) which is mounted to a Subminiature version A (SMA) connector on the circuit board as shown in Figure 3.

The omnidirectional antenna of the target node is used to boost the gain of RF power and to add some uniformity to the wave’s radiation pattern, which should eventually increase the probability of receiving packets successfully. Anchor nodes use their embedded antennas (no external antenna is attached), which cuts down setup cost and avoids additional manual work, while evaluating the EDI TestBed performance in its normal state without any modifications as compared to the work done in [17] where all sending/receiving devices are equipped with external antennas.

**Figure 4:**
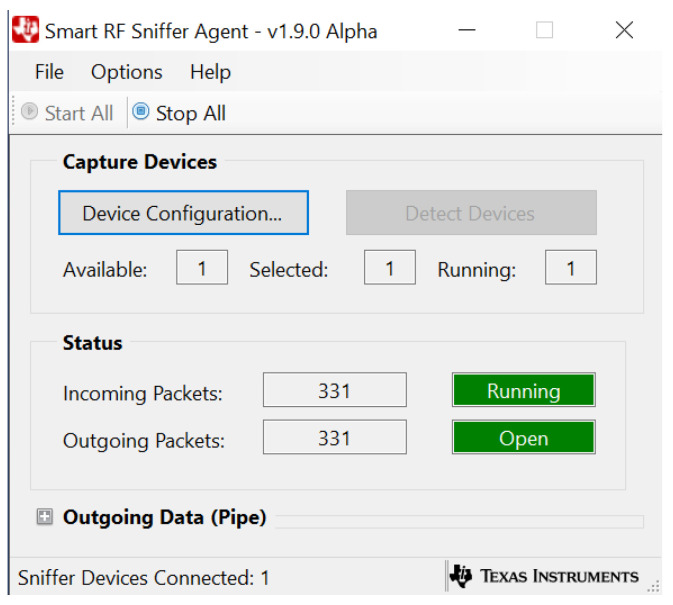
SmartRF Packet Sniffer GUI.

**Figure 5:**
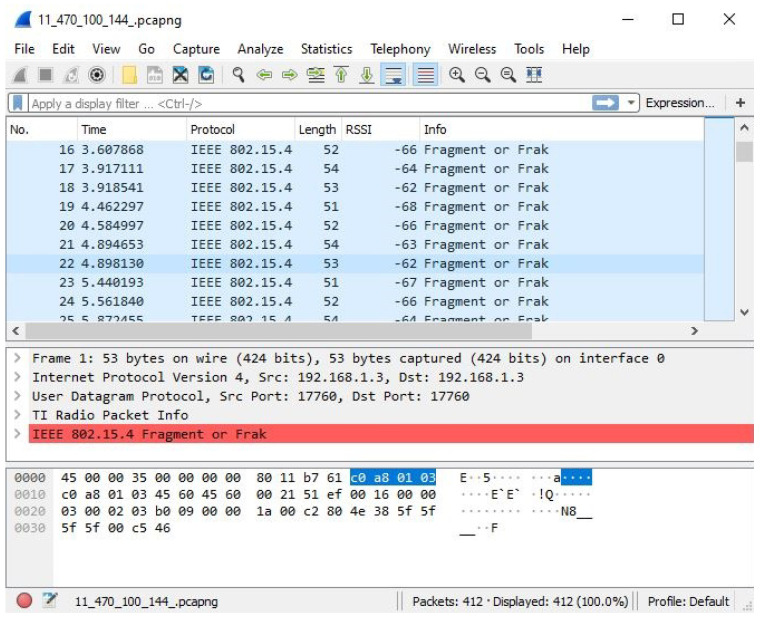
Wireshark GUI.

The SmartRF Packet Sniffer [24] software is installed on the gateway’s laptop. The software is developed by TI and it provides a graphical user interface (GUI) along with a compatible plugin with the open-source packet analyzer software Wireshark [25]. The SmartRF software is piping packets received from the CC1352R1 mobile target to the Wireshark software. By using Wireshark, the RSSI values are extracted by the radio packet information (RPI) plugin and general packets’ information are logged to the screen. The GUIs of SmartRF Packet Sniffer software and Wireshark software during the receiving process are shown in Figure 4 and Figure 5 respectively.

The “Length” column in the Wireshark interface (Figure 5) represents the size (in bytes) of received packets, with a signal strength of RSSI value indicated in the adjacent column. Received packets are distinguished based on their lengths and afterward assigned a unique NodeID value subject to the length. For example, a packet with a length of 51 bytes received from an anchor node means that the first anchor node is the sender and accordingly is assigned a NodeID of 1. Each log of packets represents the recorded data for one test/validation point at a certain power level; afterwards it is exported as a comma-separated values (CSV) file to be processed as a DataFrame using a Python script.

It is obvious that packets arriving from different anchor nodes are usually received in a random order by the target node (Figure 5), subsequently making the processing task more challenging. The ideal structure of processed packets would be chunks of sorted packets (due to their NodeIDs), each of length equal to *M* packets (*M* is total number of anchor nodes). In case a packets chunk is incomplete (contains less packets than *M*), the chunk is assumed to be corrupted and will not be further advanced by the multilateration algorithm.

**Figure 6:**
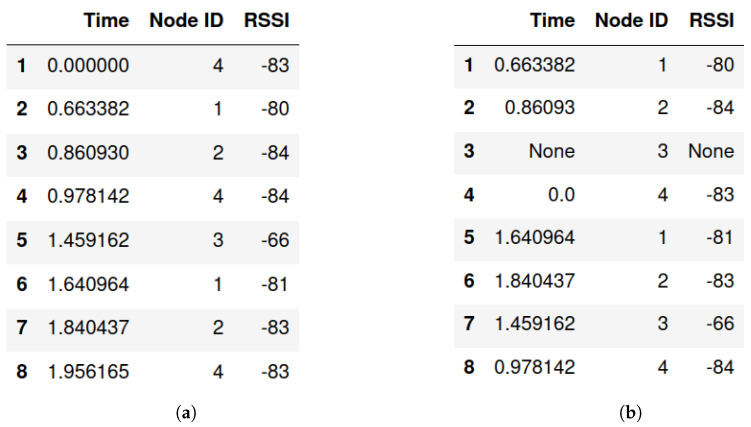
Packets processing. (**a**) Raw packets (before processing); (**b**) Processed chunks of packets.

**Figure 7:**
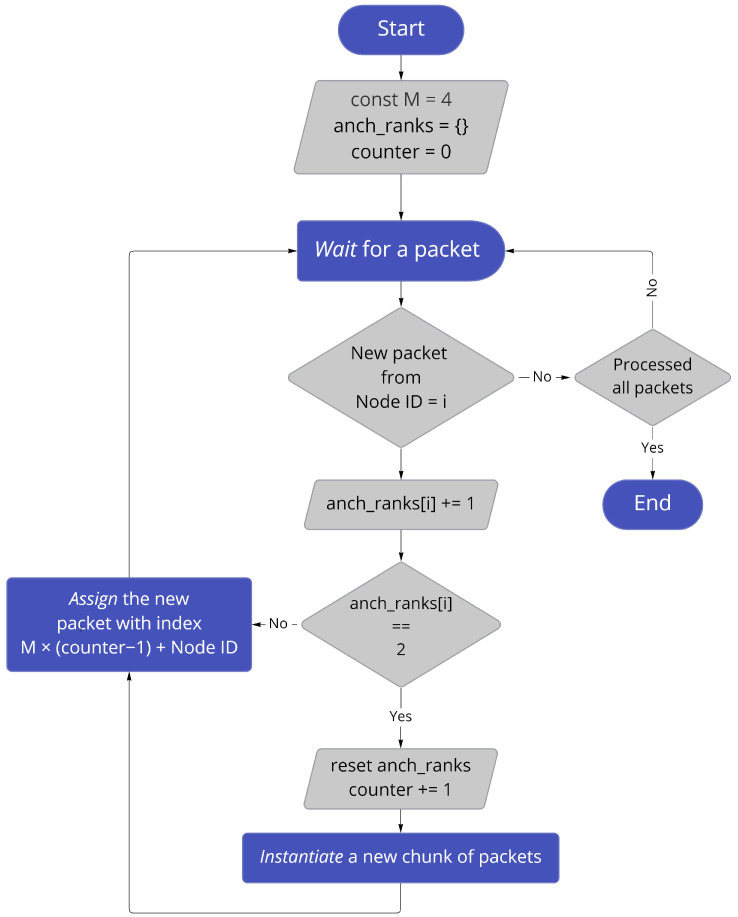
Packet sorting routine.

A sorting routine is made to facilitate the multilateration step by arranging packets and discarding incomplete chucks in advance. A numerical example for the sorting/ordering routine, assuming a configuration of 4 anchor nodes (M=4) can be described as: when the target node receives a packet from the ith anchor node (NodeID = *i*), then anchor *i* rank counter anch_ranks[i] is incremented by 1, in case the anch_ranks[i] value equals to 2. This means that a new chunk is created (increment the chunk counter and reset rank counter anch_ranks). An example of raw packets data is illustrated in Figure 6a, ordered packets structure with the first chunk being corrupted (the missed packet has an RSSI of None) is shown in Figure 6b and a flowchart of the sorting routine is illustrated in Figure 7.

### 2.3. Test and Validation Points

In order to derive the radio channel model (which tries to describe the radio wave behavior which maps RSSI values to a relative distance between the transmitter and receiver) of each anchor node antenna and to evaluate the overall performance of the localization system, a number of test points data are recorded at different RF output power levels [17]. The CC2420 radio transceiver provides 8 distinct transmission power levels, with power level 31 being the highest level and power level 3 being the lowest level in dBm. Full information about power levels can be found in the datasheet [9].

**Figure 8:**
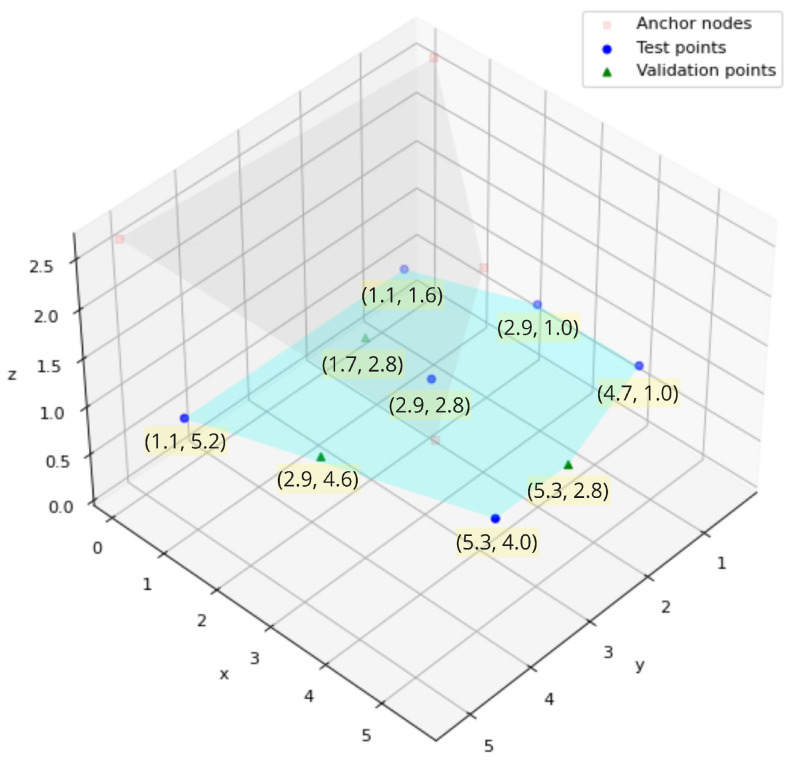
Locations of test and validation points.

Test points can be featured by positional coordinates (x,y,z) and the received RSSI value from each anchor node. Nine test points in total are recorded, and three of them are left out as validation points (not included in fitting the radio channel model) to evaluate the localization algorithm through root mean square error (RMSE) metric. Test points are distributed around the room at the same height of 1.26 m above the ground level (z=1.44 below ceiling level) as shown in Figure 8.

### 2.4. Radio Channel Model

The propagation of radio (electromagnetic) waves obeys the mechanisms of reflection, diffraction and scattering [10]. Therefore, a general characterization model for waves behavior is selected. Then this model is tweaked to match the particular conditions of the environment of interest. A simple logarithmic radio channel model, called Path loss model, is chosen to describe the RSSI at the receiver side (target node):

(1) $$P_{r}=-10\eta \log_{10}d+A$$

where:

$P_{r}$: strength of the received signal;
η: signal propagation exponent;
*d*: relative distance between sender and receiver; and
*A*: nominal transmission power at a reference distance of 1 m [17,18].

The signal propagation exponent η represents the nature of the test environment while *A* represents the radio behavior between transmitters and receiver. Both *A* and η values are obtained empirically [7]. The relative distance between transmitter and receiver can be calculated from the received power $P_{r}$ using the inverse formula of Equation (1) as:

(2) $$d=10^{\frac{P_{r}-A}{-10\eta }}.$$

#### 2.4.1. Nominal Transmission Power

**Figure 9:**
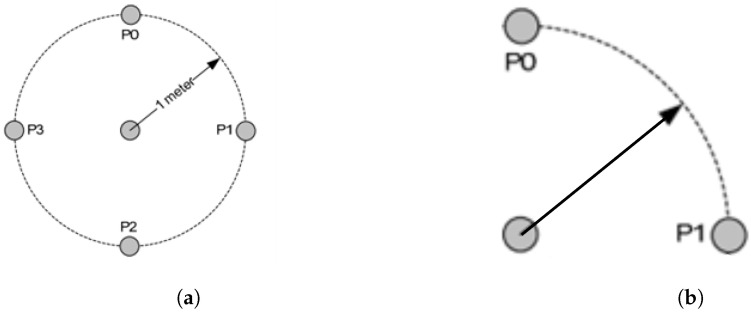
Proposed positions for estimating A coefficient. (**a**) Four calibration sets for the middle node; (**b**) Two calibration sets for corner nodes.

The nominal transmission power coefficient *A* represents the received power at a reference distance, one meter in distance from where the transmitting device is chosen. A number of packets are measured at certain positions within a circumference of the reference distance, then averaged to get an estimation of *A* [18]. The location of the workstation or anchor node affects its accessibility during the calibration step; hence four calibration sets as shown in Figure 9a can be performed to the node in the middle of the room (NodeID=3), and the rest of anchor nodes in the corners allow only two sets of packets due to their limited accessibility as shown in Figure 9b.

#### 2.4.2. Signal Propagation Exponent

For each anchor node *i*, the signal propagation exponent $\eta_{i}$ is chosen to fit the best curve matching the real Euclidean distances $d_{i}$ between the target node and the corresponding anchor node *i*, to the received signal strength $P_{r}$ measured by the target node of Equation (1) (given the nominal transmission power $A_{i}$). The logarithmic curve representing Equation (1) that best fits the test points will include the estimated value of η [17].

### 2.5. Improving System Accuracy

The accuracy of the localization system depends on the overall behavior of anchor nodes. By increasing antennas’ sensibility, any small variation of relative distance $d_{i}$ could be detected; thus a better performance of the system is obtained. A heuristic Formula (3) is used to describe the entire system sensibility in terms of signal propagation exponent η and its variability [17].

(3) $$N=\frac{\mu [\eta_{i}\left(A_{i}-RSSI_{min}\right)]}{\sigma^{2}\left[\eta_{i}\left(A_{i}-RSSI_{min}\right)\right]}$$

where

μ: mean operation between anchor nodes;
$\sigma^{2}$: variance operation between anchor nodes; and
$RSSI_{min}$: minimum detected RSSI by the corresponding anchor node.

### 2.6. Multilateration Algorithm

An estimation $\left(\hat{x},\hat{y}\right)$ of the target node’s real absolute position (x,y) could be obtained by using the multilateration algorithm, by translating estimated relative distances between anchor nodes and target node, given absolute positions of anchor nodes $\left(x_{i},y_{i}\right)$ [4]. The multilateration algorithm tries to find the estimation $\left(\hat{x},\hat{y}\right)$ within room boundaries, which minimizes the sum of the squared errors in the measured relative distances ${\tilde{d}}_{i}$. The minimization equation is given by [8,26]:

(4) $$\epsilon =\min\sum_{i=1}^{M}{\left|\sqrt{{\left(x_{i}-\hat{x}\right)}^{2}+{\left(y_{i}-\hat{y}\right)}^{2}}-{\tilde{d}}_{i}\right|}^{2}.$$

The minimization problem of Equation (4) can be solved by using least squares estimator by first converting the problem into a linear system of equations. The square of relative distance $d_{i}$ between anchor node *i* and the target node is:

(5) $$d_{i}^{2}={\left(x_{i}-x\right)}^{2}+{\left(y_{i}-y\right)}^{2}$$

where

$\left(x_{i},y_{i}\right)$: $i_{th}$ anchor node positional coordinates; and
(x,y): target node positional coordinates [26].

By taking the first node (i=1) as a reference node, subtracting Equation (5) of other nodes (i=2,3,4,...,M) from the reference node (i=1) equation, a system of linear equations is built as

(6) $$A.\bar{x}=\tilde{b}$$

where

(7) $$A=\left[\begin{array}{cc} 2(x_{1}-x_{2}) & 2(y_{1}-y_{2})\\ \vdots & \vdots \\ 2(x_{1}-x_{N}) & 2(x_{1}-x_{N}) \end{array}\right],\bar{x}=\left[\begin{array}{c} \hat{x}\\ \hat{y} \end{array}\right]$$

and

(8) $$\tilde{b}=\left[\begin{array}{c} \left(\tilde{d_{1}^{2}}-\tilde{d_{2}^{2}}\right)-\left(x_{1}^{2}-x_{2}^{2}\right)-\left(y_{1}^{2}-y_{2}^{2}\right)\\ \vdots \\ \left(\tilde{d_{1}^{2}}-\tilde{d_{N}^{2}}\right)-\left(x_{1}^{2}-x_{N}^{2}\right)-\left(y_{1}^{2}-y_{N}^{2}\right) \end{array}\right].$$

The estimated positional coordinates of the target node $\bar{x}$ can be obtained by using the LSE method as $\bar{x}={\left(A^{T}A\right)}^{-1}A^{T}\tilde{b}$ [8].

To improve the quality of the measurements, weights are assigned to each estimated distance due to its value, which turns the least squares estimation into a weighted least squares (WLS) estimation. The set of $\frac{1}{2}$, $\frac{1}{4}$, $\frac{1}{8}$ and $\frac{1}{16}$ weights are used by [17] to form the weights matrix *W*. The highest weight is assigned to the strongest signal (shortest measured relative distance ${\tilde{d}}_{min}$), as it tends to be more trustworthy. The weights matrix *W* is a bit similar to the the S matrix proposed in [26], with a modification of variances to be constants and off-diagonal elements to be zeros. The weights matrix *W* is described as:

(9) $$W=\left[\begin{array}{cccc} w_{1}+w_{2} & 0 & \cdots & 0\\ 0 & w_{1}+w_{3} & \cdots & 0\\ \vdots & \vdots & \ddots & \vdots \\ 0 & 0 & \cdots & w_{1}+w_{N} \end{array}\right]$$

and accordingly the estimated coordinates of target node $\bar{x}$ can be obtained as $\bar{x}={\left(A^{T}WA\right)}^{-1}A^{T}W\tilde{b}$.

### 2.7. Geometry Constraints

Due to the random Doppler shift inducement of radio waves and unpredictable environmental conditions, the RSSI measurement could be faulty (extremely high/low), which does not reflect a logical distance measurement [27].

**Figure 10:**
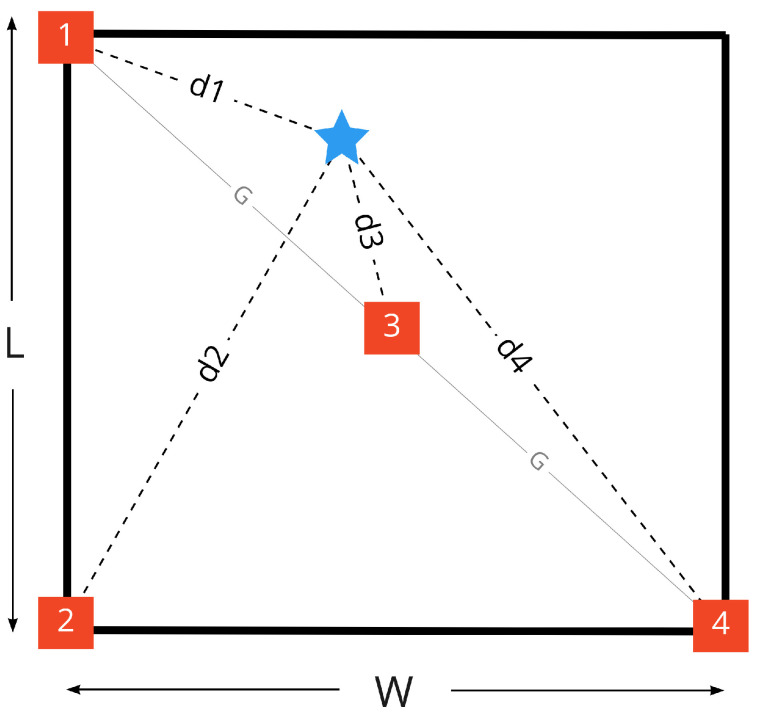
Relative distances to the target node.

Practically, measured distances between anchor nodes and target node should be within a certain range, which is defined by room dimensions and anchor node locations. This condition could help building a system of constraints to bound the distances and/or dismiss the corrupted measurements. The anchor nodes 1, 2, and 4 are assumed to be exactly at the room corners whereas node 3 is at the center of the room, this assumption is used to make the constrained system applicable and simplifies the calculations. A room layout after applying the proposed approximations, illustrating the target node as a blue star in a general position, is shown in Figure 10.

As discussed in multilateration Section 2.6, the strongest signal (or minimum measured distance) tends to be more trustworthy, accordingly it will be used as an initial distance to refine the rest of measurements by applying geometry constraints in a sequence. Distances ($d_{1},d_{2},d_{3},d_{4}$ and *G*) to be used in the following geometric constraints Algorithms 1 and 2 are illustrated in Figure 10.

After applying geometry constraints Algorithms 1 and 2, a final refinement stage is done to assure that distances $d_{1},d_{2},d_{4}$ are less than 2G distance while $d_{3}$ is less than G distance. Comparison between our methodology and [17] work is described in Table 1.
**Algorithm 1** Case 1: $d_{min}\neq d_{3}$**Require:**$-L^{2}<\alpha <L^{2}$,**Require:**$-W^{2}<\beta <W^{2}$ 1:$\alpha \leftarrow d_{2}^{2}-d_{1}^{2}$ 2:$\beta \leftarrow d_{2}^{2}-d_{4}^{2}$ 3:**if**$d_{min}=d_{1}$**then** $d_{2}^{2}=\left\{\begin{array}{c} d_{1}^{2}+L^{2},\;\mathrm{if}\;\alpha >L^{2}\\ \left|L^{2}-d_{1}^{2}\right|,\;\mathrm{if}\;\alpha <-L^{2} \end{array}\right.$ $d_{4}^{2}=\left\{\begin{array}{c} |W^{2}-d_{2}^{2}|,\;\mathrm{if}\;\beta >W^{2}\\ d_{2}^{2}+W^{2},\;\mathrm{if}\;\beta <-W^{2} \end{array}\right.$ 4:**else if**$d_{min}=d_{2}$**then** $d_{1}^{2}=\left\{\begin{array}{c} |L^{2}-d_{2}^{2}|,\;\mathrm{if}\;\alpha >L^{2}\\ d_{2}^{2}+L^{2},\;\mathrm{if}\;\alpha <-L^{2} \end{array}\right.$ $d_{4}^{2}=\left\{\begin{array}{c} |W^{2}-d_{2}^{2}|,\;\mathrm{if}\;\beta >W^{2}\\ d_{2}^{2}+W^{2},\;\mathrm{if}\;\beta <-W^{2} \end{array}\right.$ 5:**else if**$d_{min}=d_{4}$**then** $d_{2}^{2}=\left\{\begin{array}{c} d_{4}^{2}+W^{2},\;\mathrm{if}\;\beta >W^{2}\\ \left|W^{2}-d_{4}^{2}\right|,\;\mathrm{if}\;\beta <-W^{2} \end{array}\right.$ $d_{1}^{2}=\left\{\begin{array}{c} |L^{2}-d_{2}^{2}|,\;\mathrm{if}\;\alpha >L^{2}\\ d_{2}^{2}+L^{2},\;\mathrm{if}\;\alpha <-L^{2} \end{array}\right.$ 6:**end if**

**Algorithm 2** Case 2: $d_{min}=d_{3}$
 ▹$d_{min2}$: second minimum distance ▹$d_{min3}$: third minimum distance
**Require:** $-G^{2}<\gamma <G^{2}$

 1:
**if** $d_{min2}=d_{1}$ **then**
 2:    $\gamma \leftarrow d_{1}^{2}-d_{3}^{2}$

$d_{1}^{2}=\left\{\begin{array}{c} d_{3}^{2}+G^{2},\;\mathrm{if}\;\gamma >G^{2}\\ \left|G^{2}-d_{3}^{2}\right|,\;\mathrm{if}\;\gamma <-G^{2} \end{array}\right.$

 3:    $d_{min}\leftarrow d_{1}$▹ execute Algorithm 1 4:
**else if** $d_{min2}=d_{2}$ **then**
 5:    $d_{min}\leftarrow d_{min3}$▹ execute Algorithm 1 6:
**else if** $d_{min2}=d_{4}$ **then**
 7:    $\gamma \leftarrow d_{4}^{2}-d_{3}^{2}$

$d_{4}^{2}=\left\{\begin{array}{c} d_{3}^{2}+G^{2},\;\mathrm{if}\;\gamma >G^{2}\\ \left|G^{2}-d_{3}^{2}\right|,\;\mathrm{if}\;\gamma <-G^{2} \end{array}\right.$

 8:    $d_{min}\leftarrow d_{4}$▹ execute Algorithm 1 9:
**end if**



## 3. Results

In order to set up the localization system according to the procedures mentioned in the Materials and Methods Section, the first step is to calibrate anchor nodes in order to derive the radio model of each node’s antenna for the range-based localization system. The estimated values of nominal transmission power *A* [dBm] and signal propagation exponent η are listed in Table 2 and Table 3 respectively. A short description about power levels can be found in Section 2.3.

### 3.1. System Sensibility

**Figure 11:**
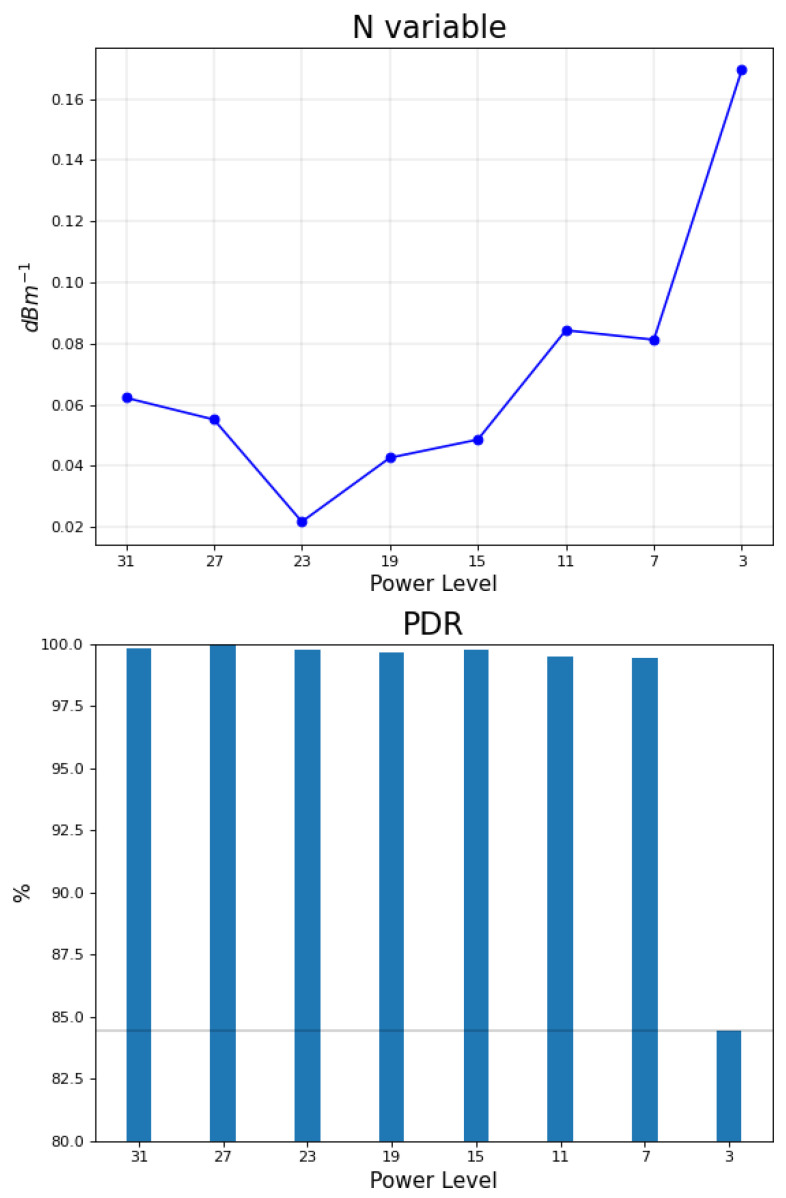
N vs. PDR for different power levels.

Given A, η (Table 2 and Table 3) and $RSSI_{min}$ values, the Formula (3) is used to figure out the optimum power level which maximizes the system sensibility according to [17], which therefore increases accuracy of the localization system as a result. The calculated values of *N* for each RF power level is illustrated in the upper side of Figure 11, while the packet delivery ratio (PDR) is illustrated in the lower side of the Figure 11.

The PDR is defined to be the ratio of number of packets received at the target node to the number of packets sent from anchor nodes. For every single test/validation point, there were approximately 100 packets sent from each anchor node, which sums up to around 400 packets in total to be received at the target node. Although the *N* variable is maximized for power level 3, the PDR plot shows that power level 3 (the lowest available RF transmission power level in the CC2420 chip) has around a 15% loss of packets, which makes the usage of the *N* value solely (without considering the corresponding PDR) at this power level unreliable because of the different (low) number of packets involved in *N* calculation for power level 3. For this reason, the power level 3 will be discarded from evaluation for the rest of the results section. The second highest *N* value is obtained for power level 11 with less than a 1% packet loss, which matches the finding of [17].

### 3.2. Performance Evaluation

The localization system is evaluated by using MSE (for test points) and RMSE (for validation points) metrics. The MSE is calculated by using Equation (10) by comparing the estimated $\left(\hat{x},\hat{y}\right)$ to the real mobile target location (x,y), then averaged by the number of samples or measurements *K* (*K* = 100 network packets for each test/validation point) [28]; the RMSE is the square root of the MSE.

(10) $$\mathrm{MSE}=\frac{1}{K}\sum_{j=1}^{K}{\left(x-\hat{x}\right)}^{2}+{\left(y-\hat{y}\right)}^{2}$$

**Figure 12:**
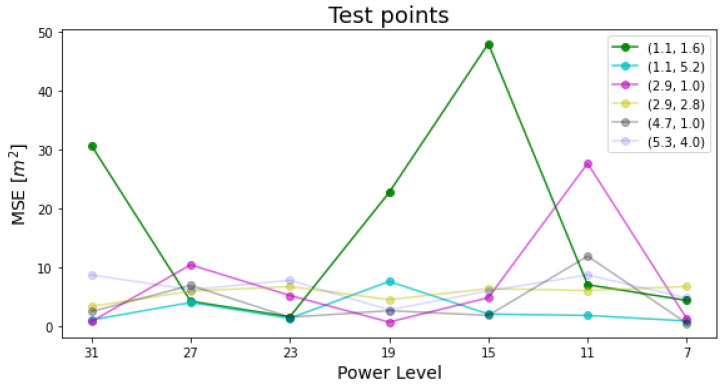
Test points MSEs.

**Figure 13:**
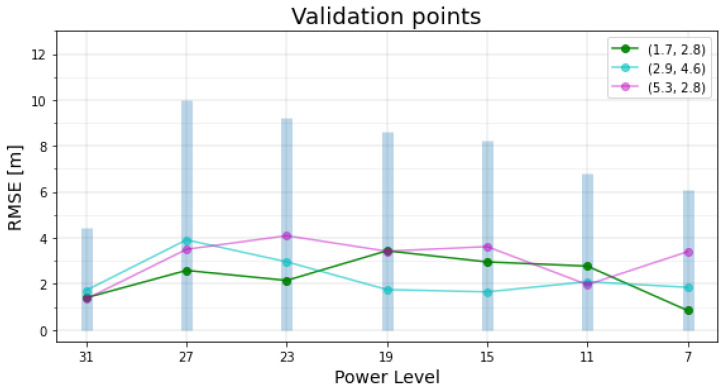
Validation points RMSEs.

The MSE and RMSE values of test and validation point measurements are illustrated in Figure 12 and Figure 13 respectively. The legend on the top-right side of each figure indicates the (x,y) positional coordinates of the point.

Vertical blue bins in Figure 13 indicates the RMSE summation for each validation point. Numerical values of the RMSEs are listed in Table 4.

## 4. Discussion

The illustrated analysis of test points data in Figure 12 shows that the majority of test points data across different power levels has an MSE range of less than 10 m, which is acceptable for such a metric taking into account operational conditions, with the exception of 4 outliers with extreme values of MSE of 20–50 m${}^{2}$ range. These outliers are caused by the continuous faulty behavior of one or more anchor node(s), which would accumulate error over samples if not instantaneously detected, then discarded (or compensated), even if the constraints of Section 2.7 are applied.

The point (2.9, 1.0) at power level 11 had a misbehaving first anchor node (NodeID=1) with a mean absolute error (MAE) of 40.4 m, whereas the rest of anchor nodes had MAE of less than 3 m. Although the power level 11 meets the condition of maximizing the system’s sensibility according to Equation (3), a single faulty node would undoubtedly ruin the localization performance. Both first and second anchor nodes (NodeID=1,2) of the peak MSE point (1.1, 1.6) at power level 15 are faulty, with MAEs of 63 and 6.8 m respectively. For the rest of the outliers, either the first node or both the first and second nodes have faulty measurements, which magnify the MSE as a result.

In order to evaluate the performance of the localization system in a stochastic environment, three accuracy classes are presumed:

1. Fair accuracy if the RMSE is less than 2 m;
2. Marginal accuracy if the RMSE is more than 2 m and less than 3 m; and
3. Low accuracy if the RMSE is more than 3 m.

The second accuracy class might turn into either first or third class in case a higher number of measurements *K* is used; hence it is called the marginal accuracy class. The power level with the least total RMSE is 31 with all validation points data belonging to the fair accuracy class, followed by two (out of three) validation points at power levels of 7 and 11. By aggregating factors of N variable, PDR (Figure 11), total RMSE (Figure 13) and accuracy classes, it could be clear that power levels 31, 7, and 11 are superior to other power levels, whereas power levels 7 and 11 could be more favourable due to their lower transmission power (lower current consumption). Although the power level 7 has lower total RMSE than level 11, the RMSE of its measurements are more disparate, which might generate a higher total RMSE value over a larger set of validation points.

According to the system sensibility Equation (3), it was expected to have the best localization performance (lowest error) at the power level meeting the maximum *N* value condition, which was validated in [17] by using a single validation point with the lowest RMSE occurring at power level 11. Three validation points were used in our experiment and it turned out that it is not the case to guarantee the best accuracy for validation points at the proposed power level (power level 11 due to Figure 11). Another factor to be considered is the PDR, for the reason that it might be misleading to rely on a power level with low PDR for evaluation; hence it is a must to assure a minimum level of PDR. According to our experiment, the minimum requirement of PDR is empirically estimated to be higher than 98% (with 2% packets loss) to obtain a balanced data set for further analysis. Regarding the utilized infrastructure of the EDI TestBed, the XM1000 devices of anchor nodes were not equipped with external antennas (embedded antennas were used), in contrast to the work of [17]. This choice was made to evaluate our testbed’s original infrastructure readiness for such an experiment. Another design choice is selecting 4 anchor nodes, sending network packets to the target node, which was made for simplicity. As a future improvement, the system could make use of more anchor nodes to mitigate the harmful effect of faulty anchor nodes; hence a better localization accuracy could be obtained. We also intend to invert the communication scheme, meaning that the target node would send one network packet at a time to anchor nodes (not the other way around), which must cancel the risk of packets collision and will bypass any routines for packets sorting (e.g., the routine described in Section 2.2). Fine tuning the transmission power levels is a beneficial feature to be added, because exploring the continuous power level range between 11–7 levels and 31–27 levels, based on our results, may reveal a better localization accuracy. The fine tuning feature is available in other IoT devices than XM1000, which demands at least a section of the EDI TestBed infrastructure to be replaced for the sake of supporting such a feature. Eventually, increasing the number of validation points may give new information about the potential reliable power level with maximum precision over a large set of measurements.

This work managed to overcome the lack of crucial information in [17] to reproduce the experiment such as: environment description (e.g., room dimensions, test points and anchor nodes positional coordinates), RSSI data analysis and processing, complete specifications of the multilateration algorithm and numerical values of calibration parameters A,η.

## 5. Conclusions

This work set out to validate the findings of [17] for investigating the influence of transmission power level on the performance of an RSSI-based indoor localization system. Reproducible implementation steps of the experiment are outlined, a geometrical constrained multilateration algorithm is developed, new factors were added to refine the system sensibility approach representation of [17], and in-depth validation results are discussed.

## Figures and Tables

**Table 1:** Comparison between our work and work done in [17].

Criterion	Our Work	Work of [17]
Radio channel model	Path loss model	
Power level selection	Equation (3)	Equation (3) and PDR
System architecture	Star topology (target node as a network sniffer)	not stated
Multilateration algorithm	GC-WLSE	probably WLSE (not clear)
Number of test, validation points	6, 3	5, 1
Anchor nodes setup	4 EDI TestBed workstations (with embedded antennas)	5 Zolertia Z1 nodes (with external antennas)
Evaluation metrics	MSE, RMSE	mean error, RMSE

**Table 2:** Nominal transmission power A of anchor nodes in dBm.

	Power Level	31	27	23	19	15	11	7	3
Anchor Node									
1		−51.02	−55.73	−54.89	−55.06	−59.95	−60.15	−64.54	−76.16
2		−46.49	−46.17	−57.08	−52.27	−53.77	−60.45	−59.22	−74.16
3		−43.99	−44.82	−46.61	−53.60	−50.27	−55.12	−58.82	−71.10
4		−40.74	−43.80	−43.25	−49.94	−49.67	−55.20	−56.74	−64.30

**Table 3:** Signal propagation exponent η of anchor nodes.

	Power Level	31	27	23	19	15	11	7	3
Anchor Node									
1		0.911	0.353	0.439	1.043	0.383	1.675	1.258	1.244
2		2.415	2.475	0.975	1.965	2.585	1.521	2.917	2.113
3		2.839	3.051	3.058	1.582	2.828	2.659	2.968	2.486
4		2.652	2.198	3.137	2.543	2.443	3.146	2.571	2.587

**Table 4:** Validation points total RMSE.

Power Level	Total RMSE [m]	Power Level	Total RMSE [m]
31	4.45	15	8.22
27	9.99	11	6.82
23	9.19	7	6.09
19	8.61	3	discarded

## Abbreviations

The following abbreviations are used in this manuscript:

GNSS	Global Navigation Satellite System
RF	Radio Frequency
RSSI	Received Signal Strength Indicator
GUI	Graphical User Interface
MSE	Mean Square Error
RMSE	Root Mean Square Error
MAE	Mean Absolute Error
LSE	Least Squares Estimation
WLS	Weighted Least Squares
GC-WLSE	Geometrical Constrained Weighted Least Squares Estimator
PDR	Packet Delivery Ratio
